# A Comparison of Four Cardiovascular Risk Assessment Instruments in Saudi Patients

**DOI:** 10.7759/cureus.7093

**Published:** 2020-02-24

**Authors:** Manar Hasabullah, Fatamah Kahtani, Tasneem Balkhoyor, Lama Al-Harbi, Abdulhalim J Kinsara

**Affiliations:** 1 Cardiology, College of Medicine, King Saud Bin Abdulaziz University for Health Sciences, Jeddah, SAU

**Keywords:** framingham risk score, systematic coronary risk evaluation, american college of cardiology/american heart association atherosclerotic cardiovascular disease risk estimator, qrisk, saudi arabia, score, risk assessment tools

## Abstract

Introduction

Several cardiovascular risk calculators are available online to measure the probability of developing cardiovascular disease (CVD) without defining the appropriate population. In the current study, four risk assessment instruments were investigated with Saudi Arabian patients with CVD to identify the instrument with the best predictability. The chosen instruments were the Framingham Risk Score (FRS), Systematic Coronary Risk Evaluation (SCORE), American College of Cardiology/American Heart Association (ACC/AHA) Atherosclerotic Cardiovascular Disease Risk Estimator, and the United Kingdom score which is called QRISK^®^.

Methods

Saudi patients, 40 years and older, with acute coronary syndrome, were recruited. Data related to age, gender, ethnicity, height, weight, systolic blood pressure, total cholesterol, high-density lipoprotein (HDL), smoking status, diabetes mellitus, rheumatoid arthritis, chronic kidney disease, atrial fibrillation, heart attack in a first-degree relative, and use of antihypertensive treatment were recorded.

Results

Out of 129 patients, the ACC/AHA had higher predictability with low risk (26.3%) and high risk (66.7%) groups. The QRISK^®^ was highly applicable (95.3%); however, the SCORE was not considered applicable (22.5%).

Conclusion

The QRISK^®^ is easy to implement and applicable in a population-based study, but the ACC/AHA is superior in predicting individuals with a high risk of CVD.

## Introduction

Cardiovascular diseases (CVD) are responsible for 46% of the mortality due to non-communicable diseases in Saudi Arabia [1]. Risk assessment instruments predict CVD and support primary prevention, but no instrument has been validated in a developing country such as Saudi Arabia. In the current study, four CVD risk assessment instruments were compared in terms of applicability and predictability, including the Framingham Risk Score (FRS), Systematic Coronary Risk Evaluation (SCORE), American College of Cardiology/American Heart Association (ACC/AHA) Atherosclerotic Cardiovascular Disease Risk Estimator, and another score frequently used in United Kingdom called QRISK®.

The FRS was the first to initiate the concept of CVD risk assessment and developed the concept of primary prevention 50 years ago [2]. According to the Canadian Cholesterol Guidelines, the FRS has been validated and is recommended in Canada since 2009 [3]. It estimates a 10-year risk for CVD by calculating the age, gender, smoking status, diabetes, high-density lipoprotein (HDL) cholesterol, total cholesterol, systolic blood pressure, and treatment of hypertension [2].

The SCORE risk chart is preferred by the European Society of Cardiology [4]. It was developed based on 12 European cohort studies and designed as two different charts for implementation in high and low-risk countries [2, 4]. The risk of CVD is calculated by measuring age, gender, systolic blood pressure, cholesterol level, and smoking status [4].

The third instrument is the ACC/AHA risk calculator, which is used for individuals from 20 years to 79 years. The AHA score is effective in detecting high risk and predicting atherosclerotic cardiovascular disease (ASCVD) as well as acute myocardial infarction. It includes age, gender, ethnicity, total cholesterol, HDL, systolic blood pressure, antihypertensive medication, diabetes status, and smoking status [5].

The QRISK® risk score is updated annually and recommended by the National Institute for Health and Care Excellence (NICE), United Kingdom [2, 6]. It incorporates age, gender, systolic blood pressure, antihypertensive medication, ethnicity, cholesterol/HDL ratio, height, weight, smoking status, chronic kidney disease (stage four or five), atrial fibrillation, diabetes, rheumatoid arthritis, and angina or heart attack in a first-degree relative younger than 60 years old. The QRISK® has been validated by comparing it against the FRS and the Scottish ASSIGN scores [6].

The four risk assessment instruments were completed with the same patient data to determine which instrument has higher applicability and predictability in the Saudi population.

## Materials and methods

A cross-sectional study was conducted with all patients admitted to the Cardiac Center, King Abdulaziz Medical City, Jeddah using a convenient sampling technique. The inclusion criteria were patients aged 40 years and older and diagnosed with the acute coronary syndrome (ACS). Patients were ineligible if they were admitted for other cardiac problems. Data were collected during an interview as well as from the electronic medical records (Best Care System). Prepared data collection sheets included age, gender, ethnicity, height, weight, systolic blood pressure, total cholesterol level, HDL cholesterol, smoking status, diabetes mellitus, rheumatoid arthritis, chronic kidney disease, atrial fibrillation, heart attack in a first-degree relative, and if any antihypertensive medication were used. The data were entered in a Microsoft Excel (Microsoft Inc., Redmond, USA) spreadsheet, and the four cardiovascular risk assessment instruments were used to calculate the risk scores.

The sample size was calculated using ClinCalc (an online calculator for medical professionals) with 80% power and 0.05 α as 129. The data were analyzed using the Statistical Package for Social Sciences (SPSS) version 21 (IBM Inc., Armonk, USA). Both descriptive and inferential statistics were done. A p-value of 0.05 with a 95% confidence interval was used to determine statistical significance. With the descriptive statistics, all categorical variables are presented as frequency and percentage and the mean ± standard deviation for all continuous variables. A Chi-square test was done to compare the demographic information with the dependent variables. All the study variables were assessed and categorized. Missing values and "not applicable" were excluded from the analyses to maintain consistency and accuracy of the study results.

The study was approved by the Institutional Review Board of King Abdullah International Medical Research Center. Written informed consent was obtained from the participants before the interview.

## Results

The study included 129 Saudi patients. The mean age was 63.1 years (± 10.7), ranging from 24 years to 91 years. The majority was male (70.5%), with 46.5% obese and 32.6% overweight. In terms of smoking status, 20.2% were smokers and 9.3% ex-smokers. The medical history indicated that the majority (79.8%) was diabetic, 29.5% had a family history of angina or heart attack in a young first-degree relative, 17.8% had chronic kidney disease, 5.4% had atrial fibrillation, the majority (78.3%) received antihypertensive treatment, and a small proportion (5.4%) was diagnosed with rheumatoid arthritis. The mean systolic blood pressure was 124 ± 19.9 mmHg, and the mean diastolic blood pressure 65.3 ± 14.4 mmHg. The mean total cholesterol was 4.1 ± 1.3 mmol/L and the HDL 0.9 ± 0.3 mmol/L (Table 1).

**Table 1:** Baseline characteristics of participants (n=129) BMI - body mass index; DM - diabetes mellitus; HTN - hypertension; SBP - systolic blood pressure; DBP - diastolic blood pressure; HDL - high-density lipoprotein; SD - standard deviation

Study variables	n (%)
Gender	
Male	91 (70.5%)
Classification of BMI	
Normal	27 (20.9%)
Overweight	42 (32.6%)
Obese	60 (46.5%)
Smoking	26 (20.2%)
DM	103 (79.8%)
Family history of heart attack/angina	38 (29.5%)
Chronic kidney disease	23 (17.8%)
Atrial fibrillation	07 (05.4%)
Treatment of HTN	101 (78.3%)
Rheumatoid arthritis	07 (05.4%)
	mean ± SD
Age in years	63.1 ± 10.7
SBP (mmHg)	124.7 ± 19.9
DBP (mmHg)	65.3 ± 14.4
Total cholesterol (mmol/L)	04.1 ± 01.3
HDL (mmol/L)	0.9 ± 0.3

The results for the four assessment instruments were: ACC/AHA 44.2%, Euro SCORE 22.5%, FRS 29.5%, and QRISK® 95.3% providing evidence that the QRISK® is the most accurate cardiovascular risk assessment instrument for the Saudi population. Classifying the sample in high and low-risk categories, the ACC/AHA detected a higher proportion of low risk (26.3%) and high risk (66.7%) patients with the SCORE detecting a higher proportion of moderate risk (55.2%) in patients. Many cases were not applicable in the four different tools: ACC/AHA (55.8%), Euro SCORE (77.5%), FRS (70.5%), and QRISK® (4.7%). Non-applicability was due to an age limitation, cholesterol level, or blood pressure level (Table 2).

**Table 2:** Cardiovascular risk score according to different assessment tools (n=129) *Some subjects had more than one reason for exclusion AHA/ACC – American Heart Association/American College of Cardiology; FRS – Framingham Risk Score

Risk classification	AHA/ACC: n (%)	Euro-SCORE; n (%)	FRS; n (%)	QRISK®; n (%)
Cardiovascular risk level applicability	57 (44.2%)	29 (22.5%)	38 (29.5%)	123 (95.3%)
Low risk (LR)	15 (26.3%)	4 (13.8%)	6 (15.8%)	16 (13.0%)
Moderate risk (MR)	4 (7.0%)	16 (55.2%)	13 (34.2%)	30 (24.4%)
High risk (HR)	38 (66.7%)	9 (31.0%)	19 (50.0%)	77 (62.6%)
Nonapplicable	72 (55.8%)	100 (77.5%)	91 (70.5%)	6 (4.7%)
Excluded by:				
Age limitation	10 (7.7%)	0 (0.0%)	2* (1.5%)	6 (4.7%)
Cholesterol level	62 (48.0%)	65* (50.4%)	89* (68.9%)	0 (0.0%)
Blood pressure level	0 (0.0%)	50* (38.7%)	1* (0.7%)	0 (0.0%)

The relationship between the sociodemographic characteristics of the sample and the four assessment instruments is displayed in Table 3. Based on the results, gender is not statistically significant, but age was statistically significant with the SCORE (X^2^=9.103, p=0.011), with patients older than 60 years at a higher risk and the FRC (X^2^= 6.348, p=0.042). Body mass index (BMI) was not statistically significant for all four instruments, but smoking was statistically significant with the ACC/AHA (X^2^ = 12.117, p=0.017), notably a higher risk was found for the non-smoker group. Diabetes mellitus (DM) was statistically significant with the FRS (X^2^=9.402, p=0.009) with diabetic patients at a higher risk.

**Table 3:** Relationship between socio demographic factors and CVD assessment tools (n=129) AHA/ACC – American Heart Association/American College of Cardiology; FRS – Framingham Risk Score; LR - low risk; MR - moderate risk; HR – high risk; BMI – body mass index; DM – diabetes mellitus; HTN - hypertension; CVD - cardiovascular disease † Not applicable were excluded from the comparison P-value has been calculated using Chi-square test ** Significant at p≤0.05 level

Factor	AHA/ACC †			SCORE †			FRS †			QRISK® †		
	LR (%)	MR (%)	HR (%)	LR (%)	MR (%)	HR (%)	LR (%)	MR (%)	HR (%)	LR (%)	MR (%)	HR (%)
Gender												
Male	46.7%	75.0%	73.7%	25.0%	62.5%	88.9%	50.0%	46.2%	73.7%	75.0%	66.7%	75.3%
Female	53.3%	25.0%	26.3%	75.0%	37.5%	11.1%	50.0%	53.8%	26.3%	25.0%	33.3%	24.7%
P-value	0.160			0.076			0.249			0.652		
Age group in years												
≤60 years old	46.7%	0	44.7%	100%	43.8%	11.1%	50.0%	76.9%	31.6%	25.0%	50.0%	39.0%
>60 years old	53.3%	100%	55.3%	0	56.2%	88.9%	50.0%	23.1%	68.4%	75.0%	50.0%	61.0%
P-value	0.208			0.011 **			0.042 **			0.248		
Classification of BMI												
Normal	0	0	26.3%	25.0%	25.0%	33.3%	33.3%	15.4%	31.6%	12.5%	13.3%	24.7%
Overweight	40.0%	75.0%	26.3%	0	25.0%	11.1%	16.7%	38.5%	26.3%	31.2%	36.7%	31.2%
Obese	60.0%	25.0%	47.4%	75.0%	50.0%	55.6%	50.0%	46.2%	42.1%	56.2%	50.0%	44.2%
P-value	0.069			0.742			0.784			0.625		
Smoking												
Yes	0	0	23.7%	0	12.5%	0	33.3%	15.4%	26.3%	0	16.7%	27.3%
No	93.3%	50.0%	68.4%	100%	87.5%	77.8%	66.7%	76.9%	57.9%	93.8%	76.7%	62.3%
Ex-smoker	06.7%	50.0%	07.9%	0	0	22.2	0	07.7%	15.8%	06.2%	06.7%	10.4%
P-value	0.017 **			0.182			0.676			0.105		
DM												
Yes	66.7%	100%	76.3%	75.0%	75.0%	77.8%	16.7%	61.5%	84.2%	81.2%	76.7%	80.5%
No	33.3%	0	23.7%	25.0%	25.0%	22.2%	83.3%	38.5%	15.8%	18.8%	23.3%	19.5%
P-value	0.379			0.987			0.009 **			0.893		
History of angina												
Yes	33.3%	25.0%	42.1%	50.0%	37.5%	33.3%	0	23.1%	36.8%	31.2%	20.0%	32.5%
No	66.7%	75.0%	57.9%	50.0%	62.5%	66.7%	100%	76.9%	63.2%	68.8%	80.0%	67.5%
P-value	0.710			0.848			0.192			0.437		
Treatment of HTN												
Yes	86.7%	75.0%	73.7%	100%	81.2%	77.8%	66.7%	69.2%	57.9%	81.2%	76.7%	77.9%
No	13.3%	25.0%	26.3%	0	18.8%	22.2%	33.3%	30.8%	42.1%	18.8%	23.3%	22.1%
P-value	0.594			0.602			0.793			0.937		

## Discussion

In the current study, the FRS, SCORE, QRISK®, and ACC/AHA risk scores were compared to identify the most applicable instrument to predict CVD risk in Saudi patients admitted with a cardiac event.

QRISK® was the most applicable instrument for the combined sample. Of the four tools, the ACC/AHA was most applicable to identify patients at high risk. Similar studies were conducted in different countries. A study in India compared three scores, the FRS, ACC/AHA & the World Health Organization (WHO) scores. Higher risks were measured in the FRS and ACC/AHA instruments (61.7% and 69.8%, respectively) compared to the WHO score [7]. In Northern Iran, the ACC/AHA score was the highest (12.96% in men and 5.87% in women) compared to lower rates for the SCORE and FRS [8]. A third study compared three risk estimators (ACC/AHA, FRS and WHO) to identify a guide for the initiation of statin therapy for the primary prevention of CVD. The ACC/AHA scored 50.2% and was considered most applicable compared to 16.9% for the FRS and 15.2% for the WHO risk chart, a finding supporting the current study [9].

We recommend the development of a Saudi specific instrument based on the variables with the highest contribution in the results of our population, including age, gender, diabetes, blood pressure level, use of antihypertensive medication, family history of the first-degree relative with angina, smoking status, BMI), and cholesterol level. Arterial fibrillation and rheumatoid arthritis are not significant. The level of physical activity may play a role. A flexible age range is recommended to include the high-risk age group (80 years and above) as a high proportion of the participants in the current study was in this age group. The ACC/AHA score has an age limitation of 79 years and the QRISK® - 84 years. In addition, two-thirds of the sample, admitted due to ACS, was male and gender should also be included. The variables used in the assessment tools did not include ex-smokers or the type and duration of diabetes. The range of blood pressure levels is limited, causing some of the exclusions in the statistical analysis. The SCORE, for example, excludes patients with systolic blood pressure above or below 120-180 mmHg. Similarly, cholesterol levels in some patients were low and outside the range. Statins were prescribed for most of the sample; consequently, their cholesterol levels were controlled. The ACC/AHA, FRS and SCORE risk scores limited the level of cholesterol for inclusion to not less than 3.6-4 mmol/L. In addition, not all the instruments included BMI, and a high proportion (46.5%) of the sample had a BMI >30 mm/Kg^2^ (obese). BMI should be considered for the adjusted instrument. Lastly, a family history of first-degree relatives with angina was found to be less relevant.

The small sample size is a limitation of the current study as well as using a cross-sectional design. However, a definite relationship between the calculators and cardiac events was established. This paper addressed an important issue of the validity of multiple validated risk scores to different ethnic groups and populations. The idea used to tailor risk scores to some ethnic group and apply risk factors for them, especially using the riskiest patients with acute coronary syndrome which is a known high-risk group.

## Conclusions

QRISK® is the most applicable cardiovascular risk assessment instrument for the Saudi population and the ACC/AHA the best at predicting high-risk individuals. The instruments are not totally applicable to the Saudi population and variables that would improve the applicability to the Saudi population have been identified. Additional research with large sample size, including primary health care patients, is recommended.
